# Clot time ratio (CTR) and treatment outcomes in Apixaban-treated atrial fibrillation patients

**DOI:** 10.1038/s41598-024-57648-0

**Published:** 2024-03-21

**Authors:** Liselotte Onelöv, Mojca Božič-Mijovski, Alenka Mavri

**Affiliations:** 1 Nordic Biomarker, Ebbegatan 9, 582 13 Linköping, Sweden; 2 Department of Vascular Diseases, University Medical Centre Ljubljana, Ljubljana, Slovenia

**Keywords:** Atrial fibrillation, Laboratory techniques and procedures

## Abstract

There are clinical situations where information about the anticoagulant effects of Apixaban could be useful. Specialised methods for measuring Apixaban concentrations are not available at all medical laboratories while methods for measuring the functional effects of Apixaban, using clot time ratio (CTR), can be performed in most medical laboratories around the clock using well-established measurement procedures. The aim of this study was to investigate CTR in trough and peak samples during Apixaban treatment of atrial fibrillation and to correlate the findings to bleeds and thrombotic events. Three trough- and three peak samples from 61 patients (31 on Apixaban 5 mg twice daily and 30 on Apixaban 2.5 mg twice daily) were analysed with MRX PT DOAC. Patients were followed for 30 + /-15 months, and bleeds and thrombotic events were documented. The effect of Apixaban could be measured with MRX PT DOAC and there was a statistically significant difference between CTR in trough samples compared to peak samples (*p* < 0.001). A total of 21 patients suffered bleeds during follow-up; two patients suffered major bleeds, and 19 suffered minor bleeds. Patients with major bleeds had both mean peak- and mean trough CTR above the respective first to third quartile (Q1–Q3) range. Four patients suffered thromboembolic events. Generally, the peak CTRs were below or in the lower end of the peak Q1–Q3 for these patients. The new test MRX PT DOAC can be used to measure the effect of Apixaban during the treatment of atrial fibrillation. High mean peak- and mean trough CTR were seen in 2 patients with major bleeds, and low peak CTR was seen in 4 patients with thromboembolic events.

## Introduction

As the prescription of direct oral anticoagulants (DOACs) is increasing, there is also an increased need for a simple and accurate laboratory test for plasma DOAC quantification. There are clinical situations where information about the anticoagulant effects of DOACs is needed: trauma, emergent surgery, neuraxial anaesthesia, and acute stroke^1^. Chromogenic Anti-FXa, or the gold standard liquid chromatography with tandem mass spectrometry (LC–MS/MS) methods for measuring DOAC plasma concentration are not available in all medical laboratories limiting the use of these tests to specialized laboratories^2^. The newly developed prothrombin (PT) DOAC test designed for measuring the functional effects of DOACs^3,4^ can, on the other hand, be available around the clock on any coagulation analyser, providing rapid results in cases of emergency.

Apixaban, one of the DOACs, is commonly prescribed in clinical practice for stroke prevention in atrial fibrillation. The recommended standard dose is 5 mg twice daily, with the option to lower the dose to 2.5 mg twice daily in patients with at least two of the following criteria: ≥ 80 years of age, body weight ≤ 60 kg, or creatinine concentration > 133 μmol/L. There is no recommendation for routine measurements of Apixaban concentration or established therapeutic intervals, but expected plasma level ranges have been determined in both clinical trials^5^ and in real-life studies^6–9^. The Apixaban plasma levels reported in these studies have a large, up to ten-fold, concentration span. Some of the studies^7,8,10^ also investigated intra-individual variations, and the average coefficient of variation (CV) ranged between 18 and 29% at the trough and 15–22% at peak for Apixaban 5 mg, and between 15 and 30% at the trough and 14–20% at peak for Apixaban 2.5 mg. Although the intra-patient variation of Apixaban plasma levels can vary up to 5x, few studies have investigated the possible correlation between Apixaban plasma levels at trough or peak with clinical outcomes such as bleeding or thromboembolic events.

This study aims to evaluate the new PT DOAC test to measure the effect of Apixaban at trough and peak, and to evaluate association between PT DOAC expressed in Clot Time Ratio CTR and clinical outcomes in patients with atrial fibrillation treated with Apixaban.

## Results

The patient groups Apixaban 5 and Apixaban 2.5 were compared with respect to demographics. Significant differences were seen for age, body weight, creatinine concentration, CrCl, CHA_2_DS_2_VASc and HAS-BLED score, while sex, history of arterial hypertension, diabetes mellitus, heart failure, ischemic heart disease, peripheral artery disease did not differ between the patient groups (Table 1^7^).

**Table 1:** Characteristics of the patients with atrial fibrillation on Apixaban. Values presented as mean (range) or count (%). CrCl creatinine clearance estimated by the Cockcroft-Gault equation. p-value is for comparison between doses.

	Either dose N = 61	Apixaban 5 N = 31	Apixaban 2.5 N = 30	Apixaban 5 vs Apixaban 2.5 p-value
Sex, female/male	36/25	15/16	21/9	0.146
Age (years)	78 (62–91)	73 (62–85)	83 (77–91)	< 0.001
Body weight (kg)	76 (45–117)	82 (50–117)	69 (45–102)	< 0.001
Creatinine (μmol/L)	90 (52–153)	82 (52–129)	98 (57–153)	0.011
CrCl (mL/min)	62 (20–147)	78 (46–147)	46 (20–89)	< 0.001
Arterial hypertension, N (%)	52 (85)	26 (84)	26 (87)	1.000
Diabetes mellitus, N (%)	10 (16)	6 (19)	4 (13)	0.731
Chronic heart failure, N (%)	17 (28)	5 (16)	12 (40)	0.073
Peripheral artery obstructive disease, N (%)	3 (5)	1 (3)	2 (7)	0.612
Ischemic heart disease, N (%)	15 (25)	8 (26)	7 (23)	0.942
Cerebrovascular insult, N (%)	10 (16)	4 (13)	6 (20)	0.508
CHA_2_DS_2_VASc score 0–1	21	19	2	< 0.001
CHA_2_DS_2_VASc score 2	15	4	11	
CHA_2_DS_2_VASc score > 2	25	8	17	
HAS-BLED score 0	7	7	0	0.021
HAS-BLED score 1	42	19	23	
HAS-BLED score 2	12	5	7	

Only 10 (33%) patients on A2.5 fulfilled the manufacturer recommendation for the apixaban dose reduction. The other patients had only one criterion: age ≥ 80 years in 17 patients, body weight ≤ 60 kg in 2 patients). One patient received a reduced dose due to concomitant dual antiplatelet treatment.

PT DOAC was determined in 180 trough and 179 peak samples (Table 2). CTR was higher in peak samples than in trough samples (*p* < 0.001).

**Table 2:** Comparison of the effect of Apixaban expressed in clot time ratio (CTR) measured in 3 trough and three peak samples. Number of samples (N), median, range (min–max) are presented for each sampling, and the average coefficient of variation (CV) between them are shown.

	Trough 1	Trough 2	Trough 3	ANOVA p	Average inter-individual CV (%)	Peak 1	Peak 2	Peak 3	ANOVA p	Average inter-individual CV (%)
**Apixaban 5**										
N	31	30	30	0.16	31	31	30	29	0.039*	31
Median	1.33	1.31	1.28		4.8	1.7	1.54	1.61		6.9
Min–max	1.09–2.17	1.13–1.74	1.01–1.88		1.33–13.36	1.35–2.89	1.23–2.32	1.12–2.62		1.18–15.03
**Apixaban 2.5**										
N	30	30	29	0.486	30	30	30	29	0.12	30
Median	1.29	1.25	1.25		3.5	1.48	1.43	1.42		4.3
Min–max	1.05–2.09	1.03–2.16	1.06–1.93		0.00–13.61	1.09–2.44	1.09–2.77	1.13–2.29		1.18–16.53

*A post-hoc pairwise multiple comparison procedure (Bonferroni -t-test) revealed that the difference was between peak sampling 1 and peak sampling 2 (*p* = 0.034).

Average CTR at trough or peak was not associated with any of the following patient characteristics: age, sex, body weight, creatinine, CrCl, CHA_2_DS_2_VASc score, HAS-BLED, arterial hypertension, diabetes, chronic heart failure, peripheral artery obstructive disease, ischemic heart disease, or cerebrovascular insult, data not shown.

Average trough CTR did not differ between Apixaban 5 vs. Apixaban 2.5 (*p* = 0.124), however, average peak CTR was significantly higher in Apixaban 5 compared to Apixaban 2.5 (*p* = 0.004).

**Figure 1:**
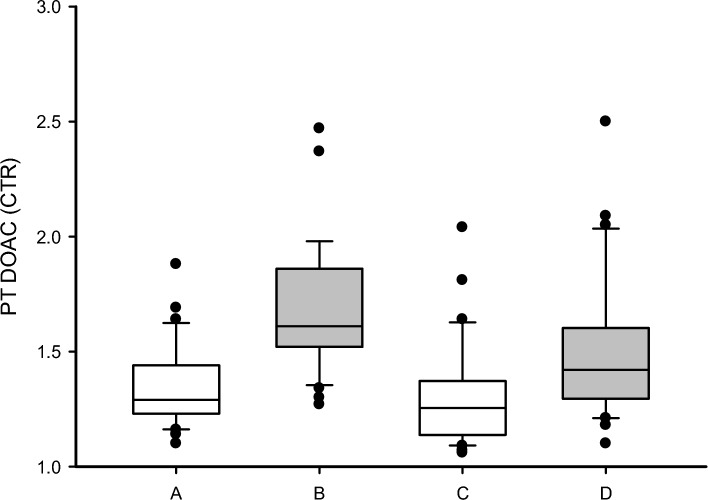
In the graph, box plots are shown to compare the distribution of mean CTR in trough samples (**A**, **C**) and peak (**B**, **D**) measured in plasma samples from patients treated with Apixaban 5 (**A**, **B**) and with Apixaban 2.5 (**C**, **D**) using MRX PT DOAC. The bottom and top of the box represent the first and third quartiles (Q_1_, Q_3_), while the band inside represents the median (second quartile). CTRs outside of the Q_1_-Q_3_ range are shown by dots. Mean CTR is significantly higher at peak than at trough for both doses.

The average CTR trough first to third quartile range (T_Q1–Q3_) for patients on Apixaban 5 was 1.22–1.45, while the average peak first to third quartile range (P_Q1–Q3_) for the same patients was 1.48–1.77, i.e., no overlap was seen between the trough and peak ranges (Fig. 1). The ranges are illustrated by box A and B in Fig. 1.

The average CTR T_Q1–Q3_ for patients on Apixaban 2.5 was 1.16–1.37 while the average P_Q1–Q3_ for the same patients was 1.29–1.59. Hence, there was an overlap between T_Q1–Q3_ and P_Q1–Q3_ for these patients (Fig. 1, box C and D).

CTR at trough samplings did not vary between the three timepoints for either Apixaban 5 or for Apixaban 2.5 (Table 2). Likewise, there was no variation in CTR at the different peak sampling timepoints for Apixaban 2.5. There was, however, a significant difference in peak CTR for Apixaban 5 patients (ANOVA *p* = 0.039, Table 2). A post-hoc pairwise multiple comparison procedure (Bonferroni-t-test) revealed that there was a difference between peak 1 and peak 2 measurements (*p* = 0.034). Further investigation revealed that the patient with the largest difference between peak 1 and peak 2 measurements caused this difference.

There was no statistically significant difference between CV for intra-individual measurements of CTR at trough (median CV 3.9%) vs. at peak (median CV 5.0%) (*p* = 0.068).

The interindividual CV for Apixaban 2.5 was lower (median CV 3.5% at the trough and 4.3% at peak) than CV for Apixaban 5 samples (median CV 4.8% at the trough and 6.9% at peak) at both peak and trough (Table 2, Mann–Whitney Rank Sum Test *p* = 0.028 and *p* = 0.015, respectively).

There was no difference in apixaban concentrations or CTR between patients who fulfilled criteria for reduced dose and those who did not (median with first to third quartile: 87 (67–122) vs 84 (66–110) ng/mL, *p* = 0.64 for average trough levels and 204 (154–255) vs 165 (143–209) ng/mL, *p* = 0.24 for average peak levels). CTR was 1.24 (1.14–1.42) and 1.28 (1.14–1.37), *p* = 0.60 in patients who fulfilled criteria for reduced dose vs those who did not.

**Figure 2:**
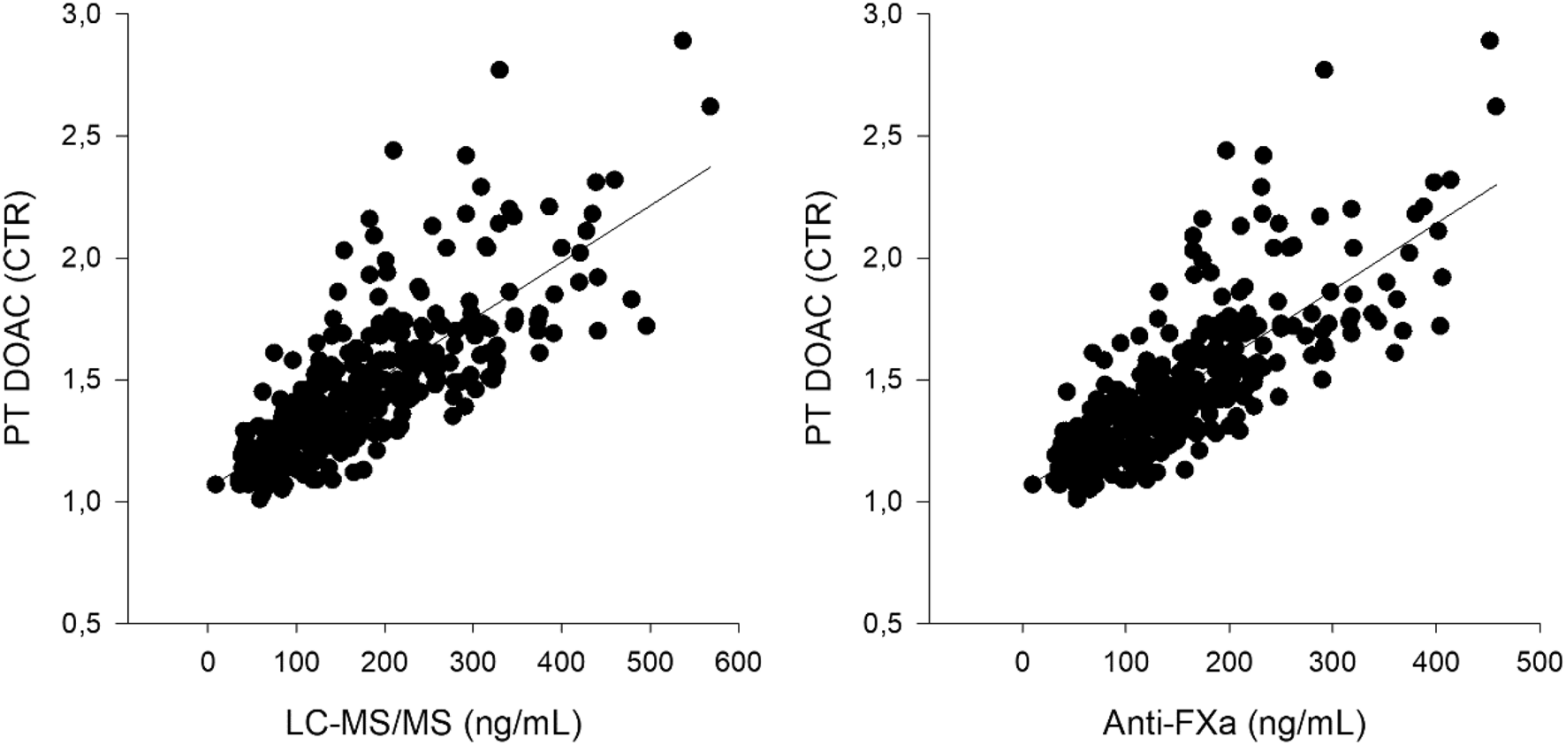
Correlations between PT DOAC (CTR) and apixaban concentration measured with LC–MS/MS (ng/mL) in (**A**) and with Anti-Xa (ng/mL) in (**B**).

There was a significant correlation between CTR, and the plasma levels determined with either LC–MS/MS (r = 0.772, *p* < 0.001) or anti-Xa (r = 0.783, *p* < 0.001) (Fig. 2). CTR also correlated significantly, but weakly, with aPTT (r = 0.332, *p* < 0.001) and PT (r = 0.329, *p* < 0.001).

**Figure 3:**
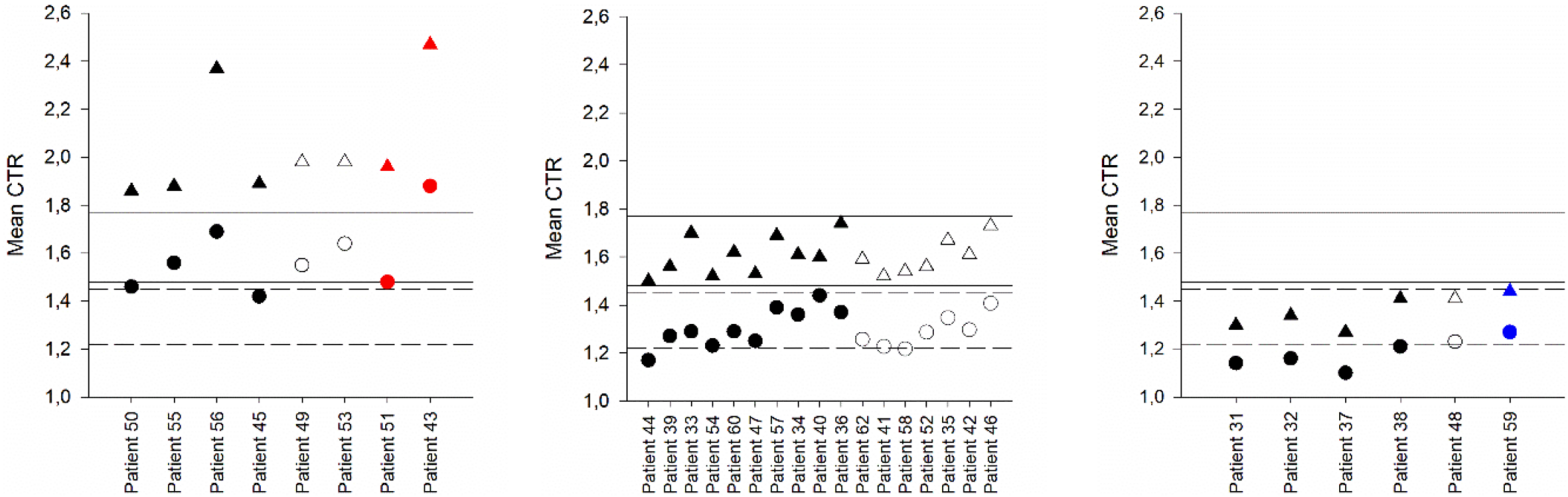
Mean CTR at trough and peak for 31 patients on Apixaban 5. Trough CTR is shown with circles and peak CTR with triangles. T_Q1-Q3_ is shown with dashed lines and P_Q1-Q3_ with solid lines. 18 patients with no bleeds or thrombosis are shown in black, one patient with thrombosis in blue, 10 patients with minor bleeds in white and two with major bleeds in red. Patients with mean peak CTR above the P_Q1-Q3_ are shown in (**A**), patients with mean peak CTR within the P_Q1-Q3_ are shown in (**B**) and patients with mean peak CTR below the P_Q1-Q3_ are shown in (**C**). Only one trough and one peak result were available from patient 50 while two were available from patient 62.

The average peak CTR was not significantly different in patients who suffered bleeds vs. those who did not (*p* = 0.216 for Apixaban 5 and *p* = 0.213 for Apixaban 2.5). However, 4 of the 8 patients (50%) on Apixaban 5 with mean peak CTR above the P_Q1-Q3_ range suffered bleeds (Fig. 3a). No major bleeds were observed for patients with mean peak CTR within the P_Q1-Q3_, while 7 out of 17 (41%) patients suffered minor bleeds (Fig. 3b). Only 1 of 6 (17%) patients with mean peak CTR below the P_Q1-Q3_ suffered a minor bleed (Fig. 3c).

Although not statistically significant the proportion of bleeds was higher in the group of Apixaban 5 patients with mean peak CTR above the P_Q1-Q3_ (50%) than in Apixaban 5 patients with the mean peak CTR within (41%) or below (17%) the P_Q1–Q3_.

Details for the 2 patients suffering major bleeds can be found in Table 3.

**Table 3:** Details for patients with major bleeds.

	Age (year) Gender Weight (kg)	Apixaban dose	CHA2DS2VASc score	HAS-BLED score	Type of bleed	Group median CTR trough/peak	CTR at trough mean	CTR at peak mean
							Trough 1, 2, 3	Peak 1, 2, 3
Patient 43	74	5 mg	1	1	Hematovitreus	1.31 / 1.61	1.48	1.96
	Female						1.49, 1.52, 1.42	1.92, 2.11, 1.85
	77							
Patient 51	82	5 mg	4	2	Epidural	1.31 / 1.61	1.88	2.47
	Female						2.17, 1.74, 1.73	2.89, 2.20, 2.31
	70							

**Figure 4:**
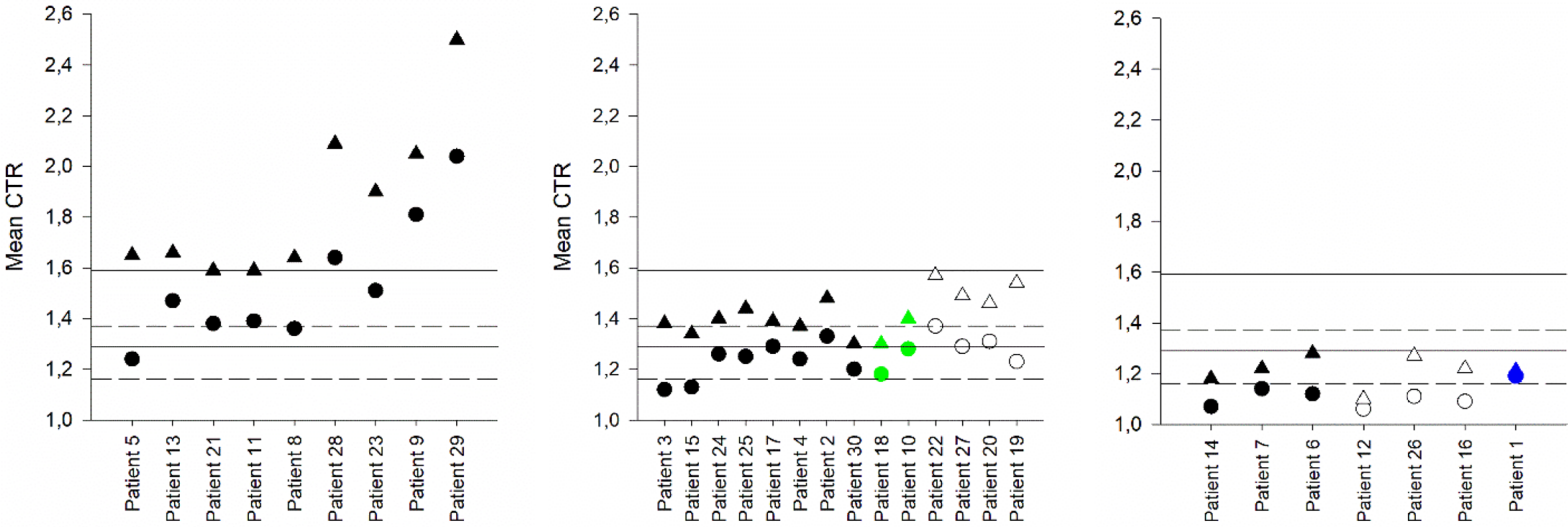
Mean CTR at trough and peak for 30 patients on Apixaban 2.5. Trough CTR is shown with circles and peak CTR with triangles. T_Q1-Q3_ is shown with dashed lines, and P_Q1-Q3_ with solid lines. 20 patients with no bleeds or thrombosis are shown in black, a patient with thrombosis in blue, two patients with both minor bleeds and thrombosis in green, and seven patients with minor bleeds in white. Patients with mean peak CTR above the P_Q1-Q3_ is shown in (**A**), patients with mean peak CTR within the P_Q1-Q3_ are shown in (**B**), and patients with mean peak CTR below the P_Q1-Q3_ are shown in (**C**). Patients 18 and 10 suffered both thrombosis and minor bleeds. Only two results were available from patient six at trough and peak respectively.

None of the nine patients on Apixaban 2.5 with mean peak CTR above the dose-specific P_Q1–Q3_, suffered bleeds (Fig. 4a). Six of the 14 (43%) patients on Apixaban 2.5 with mean peak CTR within the P_Q1–Q3_ suffered minor bleeds (Fig. 4b), while 3 of 7 (43%) patients with mean peak CTR below the P_Q1-Q3_ suffered minor bleeds (Fig. 4c).

Four patients (one on Apixaban 5 and three on Apixaban 2.5) suffered thromboembolic events; 3 NSTEMI, and 1 TIA. Peak CTR ranged between 1.14 and 1.50 for these four patients, and trough CTR ranged between 1.13 and 1.33. Trough and peak mean CTRs for patients suffering thrombosis are included in Figs. 3 and 4 and details can be found in Table 4. Generally, the peak CTRs are below or at the lower end of the dose-specific P_Q1-Q3_.

**Table 4:** Details for patients with thromboembolic events.

	Age (year) Gender Weight /kg)	Apixaban dose	CHA2DS2VASc score	HAS-BLED score	Type of thrombo-embolic event	Type of bleed	Group median CTR trough / peak	CTR at trough mean	CTR at peak mean
								Trough 1, 2, 3	Peak 1, 2, 3
Patient 1	82	2.5 mg	3	2	NSTEMI	-	1.27 / 1.43	1.19	1.21
	Male							1.20, 1.23, 1.15	1.21, 1.29, 1.14
	78								
Patient 10	88	2.5 mg	2	1	NSTEMI	Skin	1.27 / 1.43	1.28	1.4
	Female							1.30, 1.24, 1.29	1.37, 1.42, 1.42
	59								
Patient 18	85	2.5 mg	3	2	NSTEMI	Mucosal, epistaxis	1.27 / 1.43	1.18	1.3
	Male							1.13, 1.21, 1.19	1.29, 1.29, 1.33
	85								
Patient 59	84	5 mg	1	2	TIA	-	1.31 / 1.61	1.27	1.44
	Female							1.21, 1.33, 1.26	1.36, 1.50, 1.46
	85								

Two of the four patients suffered both thrombotic events and minor bleeds. These patients have all three peak CTRs within the P_Q1–Q3_, while the two patients suffering only thrombotic events had two out of three peak CTRs below the dose-specific P_Q1–Q3_.

## Discussion

There are clinical situations where information about the anticoagulant effects of Apixaban could be useful. Determination of Apixaban plasma concentrations by either LC–MS/MS or Anti-FXa has been suggested in these situations. Still, these tests are not available at all hospitals and the concentrations vary considerably both between patients and within patients. Hence a fast and easily available test for measuring the effect of Apixaban is needed as it could contribute to patient management. For this reason, we investigated the usefulness of MRX PT DOAC (CTR) at trough and peak in 61 patients with atrial fibrillation. Peak CTR values were significantly higher than trough values, i.e., the effect of Apixaban could be measured with MRX PT DOAC. In addition, patients on the full dose of 5 mg Apixaban twice daily (Apixaban 5) had a higher effect of their medication, as measured in CTR at peak, than patients on the lower 2.5 mg Apixaban twice daily dose (Apixaban 2.5) (*p* = 0.004). Average CTR at trough or peak did not associate with any of the patient characteristics investigated in this study.

We used the interval spanning the CTRs obtained for the middle 50% of the measurements for trough and peak respectively, i.e., the range between the first and third quartile at trough and peak (T_Q1-Q3_ and P_Q1-Q3_), to evaluate CTR in relation to clinical outcome. There was no overlap between T_Q1-Q3_ and P_Q1-Q3_ for the Apixaban 5 group, while an overlap was seen for the Apixaban 2.5 group, possibly due to the lower effect obtained with the reduced dose of Apixaban. The number of patients in this study was too small to evaluate whether this was due to the reduced dose or if it had any significance for clinical outcomes.

In addition to the limited availability, LC–MS/MS and Anti-FXa also suffer from high inter- and intra-variability when determining Apixaban plasma levels in real-life patients. For MRX PT DOAC, the median CV was 4.8% at the trough and 6.9% at peak for patients on Apixaban 5 and 3.5% at the trough and 4.3% at peak for patients on Apixaban 2.5. The mean intra-individual CV Apixaban plasma level determination reported by Testa et al. using an Anti-FXa method^8^ was 23% at the trough and 22% at peak for Apixaban 5 and 15% at the trough and 14% at peak for Apixaban 2.5. Similar mean CVs were reported by Toorop et al. using an Anti-FXa method^10^ of 18% at the trough and 15% at peak for Apixaban 5 and 21% at the trough and 20% at peak for the Apixaban 2.5. The method we developed, MRX PT DOAC, had noticeably lower CV compared to the anti-Xa method.

For all patients except one, no significant differences in CTR between samplings 1, 2, and 3 were observed. Taken together, in comparison to plasma level determination of Apixaban at trough and peak with either LC–MS/MS or Anti-FXa methods, the MRX PT DOAC seems to emerge as a relatively stable parameter.

Two patients suffered major bleeds, and 19 suffered minor bleeds during the length of this study. Both patients suffering major bleeds had trough- *and* peak mean CTR above the respective Q1-Q3 range. I.e., both these patients seemed to have a higher effect of their medication than most patients, and the effect remained relatively high until the next dose. For patients like these, the MRX PT DOAC test can be an easy way to evaluate the effect of the Apixaban treatment, and it would be interesting to investigate if this is true for more patients with major bleeds. In total, 50% of patients on Apixaban 5, with mean peak CTR above the P_Q1-Q3_ suffered bleeds, while 41% of patients with mean peak CTR within the P_Q1-Q3_, and 17% of patients with mean peak CTR below the P_Q1-Q3_ suffered bleeds. Although these results need to be confirmed in larger studies, they indicate that a patient with a mean CTR higher than most patients’ CTRs, could be indicative of a risk of major bleeding.

On the other side of the scale, 4 out of 20 patients (20%) with mean peak CTR close to T_Q1_ or within the T_Q1-Q3_ suffered thromboembolic events. Using MRX PT DOAC to evaluate Apixaban’s effect in these patients indicates that they might be undertreated. As reported in^7^, these patients had lower average peak Apixaban concentration, were older, and had higher HAS-BLED scores than patients without thromboembolic events. However, other studies have not found any association between Apixaban levels and thrombosis^5,9^. On the other hand, Bhagirath found a correlation between high Apixaban plasma levels at the trough and minor bleeds, while this was not seen in our study or in the study by Limcharoen et al. Interestingly, Skornova et al. and Nosal et al. investigated the Apixaban plasma level at the time of bleeding and thrombotic events respectively, and found that patients suffering bleeds had higher Apixaban levels^11^, while patients suffering thrombosis had significantly lower Apixaban levels^12^, compared to patients without bleeds or thrombotic events. Even if the published data on the correlation of Apixaban concentration with occurrence of thrombosis or bleedings is diverse and the concentration alone might not be telling the whole story, the value of measuring the effect of Apixaban in CTR must be evaluated in larger studies before any conclusions can be drawn.

There was a significant but moderate correlation between CTR, and the Apixaban plasma levels determined with LC–MS/MS, as well as with Anti-Xa. The moderate correlation between CTR and Apixaban plasma levels was expected as MRX PT DOAC measures the effect of Apixaban and not the concentration.

The correlation between CTR and aPTT or PT (INR), was, although significant, very low. While the aPTT measures the combined function of the coagulation factors in the internal pathway, the PT (INR) measures the combined function of the vitamin K dependent coagulation factors in the external pathway. The MRX PT DOAC test, on the other hand, measures the effect of Factor Xa or FIIa inhibitors on the external pathway. It is therefore not surprising, that a correlation between these different laboratory tests exists but are very low.

There have been attempts to use PT as a surrogate for Apixaban plasma level determination with the conclusion that the use of APTT and/or PT assays to screen the anticoagulant activity of Apixaban cannot be recommended^13–15^. Different protocols for diluted PT have also been investigated and proposed to be useful^16,17^, and Unami et al. reported a formula for prediction of PT values in patients receiving the standard dose of Apixaban using body weight and CHA_2_DS_2_-VASc score^18^. In their study this formula could help stratify bleeding and thrombosis risk in patients treated with Apixaban. In comparison, the MRX PT DOAC test is as easy to run as an APTT or PT, and there is no need for additional information on patient characteristics or calculations to evaluate the result.

This study aimed to evaluate the new MRX PT DOAC test as an easy and convenient test to measure the effect of Apixaban at trough and peak. We also investigated the intra-patient variation, as well as the correlation between CTR and the occurrence of bleeds and/or thrombotic events in atrial fibrillation patients treated with Apixaban. In this study we have shown that the easily available MRX PT DOAC can be used to measure the effect of Apixaban at trough and peak in patients with atrial fibrillation with low inter- and intra-individual CV. In addition, patients with major bleeds had high mean CTR at both peak and trough, while patients suffering thromboembolic events had low peak CTR.

## Materials and methods

### Patients

Sixty-one patients, treated with Apixaban for atrial fibrillation at the Anticoagulation Clinic (University Medical Centre, Ljubljana, Slovenia), were included. Thirty-one patients received the full dose 5 mg Apixaban twice daily (Apixaban 5), and 30 patients with at least two of the following characteristics received the lower dose of 2.5 mg Apixaban twice daily (Apixaban 2.5): 80 years of age or older, body weight of 60 kg or less, or serum creatinine ≥ 133 μmol/L. The lower dose was also prescribed to frail patients and patients on antiplatelet drugs, at the treating physician’s discretion. Demographics as well as CHA_2_DS_2_VASc score, HAS-BLED and creatinine clearance (CrCl) as estimated by the Cockcroft-Gault equation were recorded.

Patients were followed for 30 ± 15 months with at least yearly visits at the clinic if there were no complications requiring more frequent visits. During the follow-up, 21 patients suffered bleedings (two major and 19 minor haemorrhages; for detailed description see reference Mavri et al.). All patients signed an informed consent agreeing to participate in the study. The Medical Ethical Committee of the Slovenian Ministry of Health approved the study, and the study was performed in accordance with the Declaration of Helsinki.

### Sampling

Blood samples were drawn from the antecubital vein into 4.5 mL vacuum tubes containing 0.11 mol/L sodium citrate (9:1 v/v) (Becton Dickinson, Vacutainer System Europe, Heidelberg, Germany). Plasma was prepared with 20-min centrifugation at 2000 × *g*, aliquoted into plastic vials, snap-frozen in liquid nitrogen, and stored at -70 °C until analysis.

Three trough and three peak samples were collected from each patient at three samplings 6–8 weeks apart. Trough samples were collected 12 + /-1.5 h after the previous Apixaban dose, while peak samples were collected 123 + /-6 min after the previous dose. One patient on the lower dose missed the final sampling, and one patient on the full dose only attended the first sampling. Patients reported that they had not missed any doses in the last week prior to the blood sampling.

#### PT DOAC

MRX PT DOAC is designed to measure the effect of DOACs on the patient coagulation and is based on two, calibrated simultaneously run prothrombin time assays, where one is DOAC sensitive and the other DOAC insensitive^4^. The result is presented as the Clot Time Ratio (CTR) which is a measure of the effect of the DOAC on the patient coagulation.

PT DOAC was analysed in 359 samples on the CS2100i coagulation analyser (Sysmex, Kobe Japan) utilising the MRX PT DOAC reagent kit (Nordic Biomarker, Umeå Sweden) according to instructions from the manufacturer. One peak sample was not analysed with MRX PT DOAC due to a lack of sample.

### LC–MS/MS, anti-Xa, aPTT and PT

LC–MS/MS was performed as previously described^19^. Anti-Xa (Innovance Heparin, Siemens, Marburg, Germany), aPTT (Pathromtin SL, Siemens, Marburg, Germany), and PT (Thromborel S, Siemens, Marburg, Germany), were analysed on the CS-2500 coagulation analyser (Sysmex, Japan).

### Statistical methods

Categorical variables are presented as counts and percentages, while continuous variables are presented as mean or median with range or first to third quartile (Q1-Q3).

The within-patient trough and peak coefficient of variation (CV) were calculated as standard deviation/average × 100 from all available trough and peak measurements of each patient.

A statistical comparison was performed by using a t-test or Mann–Whitney Rank Sum test for the continuous variables depending on if the assumption of normality was fulfilled or not, and for the categorical variables with a chi^2^ test or depending on sample size a Fisher exact test.

Correlations were analysed using Pearson product-moment correlation as well as linear regression.

One-way repeated measures ANOVA was used for the comparison of repeated samplings. A post hoc pairwise multiple comparison procedure (Bonferroni adjusted t-test) was used to further investigate differences between repeated samplings when the ANOVA result was below *p* < 0.05.

Statistical evaluations of results were done with SigmaPlot 14.0 and MS Excel. Two-sided *p* < 0.05 was considered statistically significant.

## Data Availability

The datasets generated during and/or analysed during the current study are available from the corresponding author on reasonable request.

## References

[1] Douxfils J (2021). Update of the international council for standardization in haematology recommendations for laboratory measurement of direct oral anticoagulants. Thromb. Haemost..

[2] Douxfils J (2018). Laboratory testing in patients treated with direct oral anticoagulants: A practical guide for clinicians. J. Thromb. Haemost..

[3] Lindahl TL (2017). A novel prothrombin time method to measure all non-vitamin K-dependent oral anticoagulants (NOACs). Ups J. Med. Sci..

[4] Abelius M; Theodorsson ERM., Lindahl T, *A Novel Prothrombin Time (PT) Assay ‐ A Tool for Rapid Screening of All Non‐Vitamin K‐Dependent Oral Anticoagulants (NOACs).* Thromb Haemost, 2018. **2**(S1).

[5] Bhagirath VC (2017). Apixaban-calibrated anti-FXa activity in relation to outcome events and clinical characteristics in patients with atrial fibrillation: results from the AVERROES trial. TH Open.

[6] Rosian, A.N., et al., Interindividual variability of apixaban plasma concentrations: Influence of clinical and genetic factors in a real-life cohort of atrial fibrillation patients*.**Genes (Basel)*, 2020. **11**(4). 10.3390/genes11040438 PMC7230214 32316515

[7] Mavri A (2021). Apixaban concentration variability and relation to clinical outcomes in real-life patients with atrial fibrillation. Sci. Rep..

[8] Testa S (2016). Plasma levels of direct oral anticoagulants in real life patients with atrial fibrillation: Results observed in four anticoagulation clinics. Thromb. Res..

[9] Limcharoen, S., et al., *Do Apixaban Plasma Levels Relate to Bleeding? The Clinical Outcomes and Predictive Factors for Bleeding in Patients with Non-Valvular Atrial Fibrillation.* Biomedicines, 2022. **10**(8). 10.3390/biomedicines10082001 PMC9406092 36009548

[10] Toorop MMA (2022). Inter- and intra-individual concentrations of direct oral anticoagulants: The KIDOAC study. J. Thromb. Haemost..

[11] Skornova I (2021). Direct oral anticoagulants plasma levels in patients with atrial fibrillation at the time of bleeding: A pilot prospective study. J. Cardiovasc. Pharmacol..

[12] Nosal V (2022). Plasma levels of direct oral anticoagulants in atrial fibrillation patients at the time of embolic stroke: A pilot prospective multicenter study. Eur. J. Clin. Pharmacol..

[13] Adcock DM (2013). The effect of dabigatran on select specialty coagulation assays. Am. J. Clin. Pathol..

[14] Hillarp A (2014). Effects of the oral, direct factor Xa inhibitor apixaban on routine coagulation assays and anti-FXa assays. J. Thromb. Haemost..

[15] Kitchen S (2014). Measurement of non-coumarin anticoagulants and their effects on tests of Haemostasis: Guidance from the British committee for standards in haematology. Br. J. Haematol..

[16] Letertre LR (2016). A single test to assay warfarin, dabigatran, rivaroxaban, apixaban, unfractionated heparin, and enoxaparin in plasma. J. Thromb. Haemost..

[17] Ieko M (2020). Novel assay based on diluted prothrombin time reflects anticoagulant effects of direct oral factor Xa inhibitors: Results of multicenter study in Japan. Thromb. Res..

[18] Unami N, Ise Y, Suzuki H (2020). Anticoagulant activity of apixaban can be estimated by multiple regression analysis. J. Arrhythm.

[19] Skeppholm M (2015). Clinical evaluation of laboratory methods to monitor apixaban treatment in patients with atrial fibrillation. Thromb. Res..

